# Societies with fission–fusion dynamics as complex adaptive systems: the importance of scale

**DOI:** 10.1098/rstb.2023.0175

**Published:** 2024-07-22

**Authors:** Anastasia Madsen, Shermin de Silva

**Affiliations:** ^1^ Department of Ecology, Behavior and Evolution, University of California, San Diego, CA 92093-0021, USA

**Keywords:** complex systems, complex adaptive systems, fission–fusion dynamics, dynamic systems, scale

## Abstract

In this article, we argue that social systems with fission–fusion (FF) dynamics are best characterized within a complex adaptive systems (CAS) framework. We discuss how different endogenous and exogenous factors drive scale-dependent network properties across temporal, spatial and social domains. Importantly, this view treats the dynamics themselves as objects of study, rather than variously defined notions of static ‘social groups’ that have hitherto dominated thinking in behavioural ecology. CAS approaches allow us to interrogate FF dynamics in taxa that do not conform to more traditional conceptualizations of sociality and encourage us to pose new types of questions regarding the sources of stability and change in social systems, distinguishing regular variations from those that would lead to system-level reorganization.

This article is part of the theme issue ‘Connected interactions: enriching food web research by spatial and social interactions’.

## 1. Introduction

The term ‘fission–fusion’ (FF) classically described the phenomenon of animals adjusting group size and composition in response to environmental or social cues, with the term ‘fission–fusion society’ originally being applied to a narrow subset of mammalian taxa evidencing flexibility in association patterns among individuals [1]. However, Aureli *et al.* [2] have argued that FF dynamics can be present to varying degrees in many species, broadly enabling groups to balance the costs and benefits of sociality on heterogenous landscapes. They and others model and describe aspects of spatiotemporal variation in grouping patterns in terms of socioecological drivers [1–5], which can also be studied in terms of networks [6]. For example, abundant and homogenously distributed resources can lead to denser and less modular social networks (i.e. less well-defined social groups) [7–10], whereas heterogenous distributions result in lower densities and higher modularity [7,11,12]. Predation risk also influences FF, with fusion into larger aggregations favoured under conditions with higher risk [8,13,14]. However, factors such as kinship, strength of relationships and dominance interactions can influence not only aggregation sizes but also individuals’ choice of social partners [8,15]. Unlike classical socioecological models, in which individuals are treated as essentially homogenous particles reacting to social and environmental conditions, these considerations encouraged the development of agent-based models to predict FF dynamics [2].

Sueur *et al*. [3] have gone a step further in considering FF dynamics within the context of collective behaviour, where higher rates of FF occur when individual and group needs no longer align. They especially highlight the need for individual-based models in systems where social groups are not stable or clearly defined, and where relationships among individuals are heterogenous [3]. Despite this, there continues to be a tendency among behavioural ecologists to classify taxa with FF dynamics based on supposedly species-typical characteristics of group size and composition, giving primacy to taxa that appear to show clear social boundaries (e.g. [16], table 1). This disconnect arises from a failure to recognize that FF processes may manifest differently at various *scales* and *domains* of analysis. This is a critical issue, as Levin [22] once argued, ‘the problem of relating phenomena across scales is the central problem in biology and in all of science’. This can result in gross oversimplifications of system dynamics that do not adequately capture reality. Levin [22] illustrated this with the example of a simple diffusion model for invasive species dynamics, which performed well for data on local scales but failed when scaled up as it did not account for multiple centres of spread. In the context of *social systems*, FF dynamics bridge individual decisions, social group formation and higher-level *social structures* through emergent patterns and feedback mechanisms within and across scales [22]. Fortunately, there is no need to create novel frameworks for integrating considerations of scale into the study of FF dynamics, as there is already a well-established paradigm with the tools for doing so: that of complex adaptive systems (CAS, cf. ‘complex systems’).

**Table 1.** Definitions and examples of components of CAS. We define our operative use of CAS components to apply this framework to social systems that exhibit FF dynamics.

concept	definition	parallel term(s)/concept(s)	example(s)
agent	actor in a system that moves independently and responds to a local environment according to a set of rules that can be updated as local conditions change (see autonomy, updation). Here, an individual animal in the social system	element, node	any individual organism
autonomy	agents can make individual decisions; lack of centralized control or central memory	intelligence [17]	
discontinuity [18]	a property of a complex system where patterns and processes are relatively self-contained within discrete scales of organization		
domain	a parameter space defined within an analytical category		temporal, spatial, social
endogenous factors [19]	factors that influence system dynamics from within the system, resulting in feedbacks between agent behaviour and system structure		kinship, dominance, demography, life history
exogenous factors [19]	factors that exist independently (outside) of the system and do not consist of components similar to the system (i.e. other agents of the same type)		diel and seasonal cycles, resource distributions
force	the degree of attraction/repulsion an agent feels to objects in the world according to its internal state and motivations (contrast to: rules)		attraction/repulsion to resources and conspecifics
goal-directed	oriented towards a specific outcome; responsive relative to an individual’s needs and local environmental conditions		
history	multiple past interactions between pairs of agents that establish a basis for their associations		attraction or avoidance between familiar individuals
memory	the tendency of agents to respond using rules based on past conditions; the overall tendency of the system to continue along a trajectory based on past conditions	hysteresis, path dependency	
nonlinearity	exponential relations between inputs and outputs of a system owing to (i) the ability of autonomous agents to update rules based on local conditions and (ii) feedbacks between individual actions and systemic patterns		logistic growth, alternative stable states
rules	decision-making process internal to the agent		‘find food’; if *x* then *y*
scale	a level of description in time, space or social organization that is distinct from others	aggregation [18], unit	see sections 2, 3 and 4.
scale dependence	processes and patterns occur relating to particular scales of organization		
self-organization	the process by which global system properties arise from the interactions of lower-level elements (see agent) without global control or knowledge of global patterns (see autonomy)		
social organization [20]	the grouping behaviour of agents into one or more social scales		discrete sets of nodes in a social network; community clusters [20]
social structure [20]	the set of interactions or connections between individuals		edge topology in a social network [20]
social system	a set of self-similar agents (here, conspecifics) that are structured through relations that allow them to achieve an emergent global function or pattern of organization		
state change	on a stability landscape, where a system moves to a new equilibrium	regime shift [18,21]	
switch point	the distinct transition between scales of organization where rules or patterns of the previous scale no longer apply (see scale dependence)	boundary conditions	transition between dyadic and social community scales
trigger	an event that catalyses a state change at a particular scale (e.g. a threshold effect) or transitions between scales		hormonal, ecological or demographic changes
updation	ability of agents to flexibly change rules guiding responses to local conditions	adaptation, evolution	

CAS is a multidisciplinary paradigm that seeks to explain how localized interactions among system components can result in emergent structures and dynamics [18,19,23]. Systems thinking is well-established in many fields ranging from landscape ecology [18,24] to biology [25,26] and behavioural economics [19,27]. We propose that CAS is a natural way of characterizing and studying FF processes across diverse social systems that can be extended well beyond current applications in the study of collective movements (e.g. swarm dynamics and coordinated movement) [17,28]. There are at least three reasons to apply CAS paradigms to FF dynamics. First and foremost, this approach allows us to treat social dynamics themselves as objects of study rather than pre-conceived abstractions of social groupings, which in many cases may be transient emergent states at various spatial, temporal or organizational scales. This in turn allows us to recognize and interrogate FF dynamics in taxa that do not conform to traditional socioecological models and are therefore often neglected. In doing so, it encourages us to pose different questions with respect to what drives or maintains FF dynamics than frameworks that are traditionally preoccupied with predicting group sizes under specific socioecological regimes.

### (a) Features of complex adaptive systems

Biologists and complex systems scientists regrettably use identical words to signify different things. These include terms such as ‘complexity’ itself, along with ‘intelligence,’ ‘stability,’ ‘learning,’ ‘adaptation’ and ‘evolution’ to name a few [19]. We therefore specify our own usage of common terms and offer some substitutes for those that require differentiation from general use in biology (table 1). We first disambiguate the term ‘complexity’ in the context of FF dynamics from its general use in studies of behaviour, as it can be applied in many ways [29]. The concept of ‘social complexity’ has for instance been variously defined along categorically distinct dimensions relating to the structure, quantity and quality of social relationships [20,30], but is rarely an explicit reference to CAS. ‘Complex’ societies often do entail FF dynamics in some form, for example, multi-level organization [16]. However, the term also often describes hierarchically stratified societies that demonstrate features of CAS but that do not exhibit FF dynamics, such as those of eusocial insects. The former falls within our scope, the latter does not. Studies of instantaneous collective movements involving anonymous/homogenous individuals also fall within the domain of CAS but are not under consideration here as they do not represent *social* interactions. We focus on situations in which individuals have *memory* and *history* with respect to one another.

While there is no single definition of CAS, they share certain key features that are of particular relevance with respect to social systems. They consist of *autonomous agents* (as distinguished from physical particles) that *self-organize* through localized interactions. Agents need not possess sophisticated cognition (though the possibility is not excluded), but they are *goal-directed* and follow *rules*. They are also self-similar (i.e. individuals of the same species) but not homogenous (have differing goals, needs, etc.). The ‘adaptability’ comes from two possible means of *updation* or changing the behaviour of the system. Either the agents themselves update the rules guiding their behaviour or the system as a whole changes in response to some perturbation. Phenomena at one scale may influence those at other scales. When smaller scales influence larger scales, this gives rise to *emergent* properties; when larger scales influence smaller scales this is typically thought of as *feedback*. Feedback leads to *nonlinearities* wherein some components become coupled such that small changes in parameter values lead to sudden and disproportionate changes in the system [18,29,31].

FF processes may appear to be random or else structured by the ecological and social environment [4]. Factors like resource distribution and predation risk are classically thought of as categorically distinct selective pressures. However, from the individual’s perspective, they can both be considered *forces* influencing decision-making. Specifically, they exemplify *exogenous* drivers as they are outside of the social system itself and are not typically responsive to feedback from the social system [19]. Individuals also make decisions to join or leave conspecifics as a result (or in anticipation) of social interactions, representing *endogenous* influences that occur between self-similar agents and are subject to feedback from the system itself. For example, individuals may associate with conspecifics based on endogenous forces like kinship or phenotype matching [3,32]. Social associations are then reinforced by benefits from familiar conspecifics, such as fewer aggressive interactions [33,34], increased fitness [35,36] or information sharing [37,38], leading to repeated associations between the same individuals (i.e. feedback). These considerations can drive preferences for conspecifics that extend beyond mere resource attraction or predator dilution [39–43].

The scale at which such individual decisions are made differs greatly from the scale at which global network dynamics are observed. Therefore, it is useful to explicitly recognize different organizational scales across the temporal, spatial and social domains of analysis. A given scale within one domain may map onto one or more different scales in either of the other two, therefore they are conceptually distinct despite being phenomenologically inseparable. CAS not only allows us to investigate patterns and processes within the appropriate scale but also to specifically interrogate the connections within and between scales. We discuss scale-dependent social dynamics, provide examples of how endogenous and exogenous influences contribute to them and explore patterns that do not fit into classic socioecological conceptualizations of social groups.

## 2. Temporal scale

**Figure 1.**
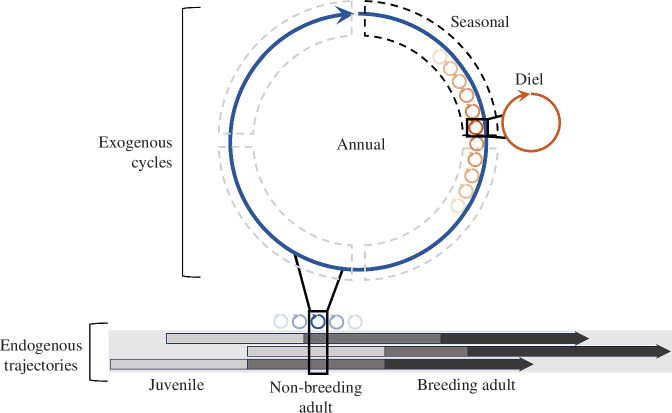
Nested temporal scales. Exogenous drivers such as annual, seasonal and diel cycles are inherently nested within each other and within endogenous temporal scales, like life trajectories. Both synchronous (e.g. annual cycle) and asynchronous temporal scales (e.g. life trajectories) introduce feedbacks and nonlinearities that result in different patterns of social organization at different scales and over the lifespan of individuals, communities and meta-communities.

There are many different biologically meaningful timescales that an organism can be tuned to, some of which may be ubiquitous and readily identifiable (e.g. exogenous *switch points*), others that may not (e.g. endogenous switch points). Exogenous switch points include diurnal, seasonal and annual cycles that are *triggered* by environmental cues such as photoperiods and temperature, while endogenous switch points include life-history stages or demographic changes (i.e. birth/death/immigration/emigration events and overall population growth/decline) that are triggered by changes within the individual or society and need not show any periodicity or synchrony. Ideally, to fully understand FF dynamics, we should investigate social structure across multiple temporal scales (figure 1), employing a CAS approach to identify scale-relevant questions and sources of feedbacks or perturbations that influence social dynamics. While system-specific features may determine the appropriate timescale of analysis, one challenge is that it is not always obvious in dynamic networks [44]. Moreover, the temporal scales that we loosely classify as short-, intermediate or long-term are not absolute, but relative to the species’ pace of life.

Short-term interactions include minute-to-minute and day-to-day decisions that individuals make to join and leave groups. Motivations such as escaping a predator (an exogenous trigger) can drive group dynamics on multiple timescales. The shortest timescales involve instantaneous decisions made during collective movement (e.g. predator-avoidance manoeuvre), but these are categorically distinct processes in comparison to the decision of whether to join or leave social groups over longer timescales. These may include a combination of exogenous and endogenous drivers. For example, Eurasian jackdaws (*Coloeus monedula*) form small, ephemeral foraging flocks during the day and large roosting flocks at night [45]; Bechstein’s bats (*Myotis bechsteinii*) are essentially solitary while foraging at night but return to the same roosting colony during the day [46]. Though roosting itself is cyclically triggered by exogenous diel cues, the decision of whom to roost with is dictated by endogenous social preferences. This is an example of a feedback mechanism because in addition to exogenous factors that draw individuals to a location at any given time, individuals also return repeatedly to where they expect to find familiar conspecifics [45,46]. We discuss further this in the context of spatial scales.

Intermediate timescales may also exhibit natural synchronous periodicity as a result of exogenous triggers such as seasons. These interact with endogenous triggers, such as the need to engage in breeding or migratory movements. Species with discrete breeding and non-breeding seasons show clear shifts in their social structure. Temperate bird species present a key example of this change: while nesting, breeding pairs generally become more closely connected to each other and more isolated from others, then individual degree and connectivity increases in winter when birds associate more broadly [39,41,45]. Migratory animals can show even more extreme changes between seasons, sometimes associating with completely different social partners and groups between breeding and non-breeding ranges [47,48]. Kinship can also influence seasonal affiliations, for example, when kin-based groups sever connections with others during periods of resource scarcity and/or higher competition and regain connections to non-kin during higher resource availability [7,32,49,50]. Exogenous switch points can show strong synchronicity, but some endogenous triggers are asynchronous within populations, such as the expression of musth (a rut-like breeding state) in male elephants (*Loxodona* spp. and *Elephas*), in which individuals shift their strategies of association as well as space use [51,52]. The coupling of interactions between exogenous and endogenous factors is important to consider because they introduce feedbacks and nonlinearities to social organization within and across seasonal scales (e.g. carry-over effects [53]).

An agent’s lifespan is a discrete and finite period, but societies typically consist of multiple overlapping generations (figure 1). Therefore, although individuals may follow broadly similar life histories from birth to death, the age structure of a population allows intergenerational interactions. Individuals change the degree and strength of their connections to particular age classes over the course of their lives. Juveniles may start with fewer connections as they associate primarily with their parents and natal associates, while older individuals acquire social affiliates over their lifetimes [7]; the reverse may be evidenced in systems where juveniles are less socially selective but grow to have fewer, stronger connections [54–58]. As a simple example of *updation*, a juvenile may follow one rule: ‘stay with parent’ The parent can fulfil more than one requirement, providing both food and safety, thus exhibiting the strongest attractive force. However, as the animal matures, hormonal changes may trigger a new life-history stage where the rule is replaced with two new rules: ‘find food’ and ‘find mates’ that are associated with different and perhaps competing goals. The parent, likewise, may initially be motivated to maintain a relationship with offspring but actively reject it once other priorities take precedence. Different age classes may play by different sets of rules [59], resulting in heterogeneity in connections at higher-order organizational scales. Even longer timescales including multiple successive generations may be necessary for observing changes in entire communities, as discussed below.

## 3. Spatial scale

**Figure 2.**
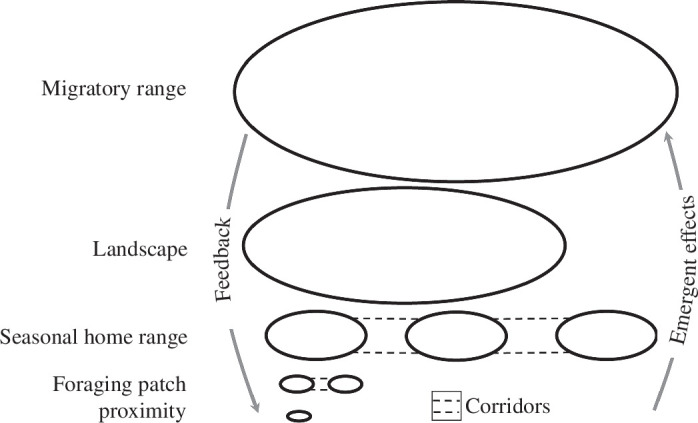
Nested spatial scales. Social (endogenous) and ecological (exogenous) processes take place within nested spatial scales that are reinforced/perturbed by feedback loops between spatial scales. Within scales, corridors allow individuals to move between areas, while other landscape features restrict movements. These spatial constraints influence both the spatial and social structure of populations as individuals move through the same spaces over time.

For virtually all species aside from (arguably) humans, there can be no such thing as social connectivity without spatial connectivity. This is true even for species that are physically capable of communication over great distances. Physical proximity is therefore the most basic spatial scale for observing and analysing FF dynamics, out of which every other pattern emerges (figure 2). Proximity may be measured across various other spatial scales such as foraging patch, home range and longer dispersal distances (figure 2). As with the temporal scale, observers may define ‘close’ proximity differently across systems [39,60,61] and often make some decisions in determining which individuals in an aggregation are socially associated, known as the ‘gambit of the group’ [62]. Regardless, the smallest spatial scales involve instantaneous movement decisions of agents based on their ability to perceive nearby conspecifics, predators and resources. Their decisions with respect to feeding, mating and movement at larger scales may differ based on the various exogenous and endogenous triggers to which individuals are responding.

Processes like habitat selection represent responses to different exogenous triggers at intermediate scales because agents are attracted to habitat types that meet their specific requirements [63]. Given the autonomy and heterogeneity of individuals, distinct areas where individuals interact most often with other sets of individuals can emerge at various spatiotemporal scales, whether foraging patches or home ranges. While larger spatial scales generally require longer timescales for individuals to traverse, there need not be a direct or consistent mapping of scales across spatial and temporal domains. Roosts and dens are examples of confined spaces to which individuals show high fidelity, structuring associations over multiple timescales [45,64]. Indeed, the formation of habits and pathways linked to particular locations can even precipitate multi-generational resource-use traditions. For instance, African forest elephants (*Loxodonta cyclotis*) appear to preferentially return to particular bais (forest clearings containing mineral deposits) despite the availability of other similar sites precisely because they are used by conspecifics, again illustrating reinforcing feedback [65,66]. Opposing forces like affiliation and aggression can act simultaneously, attracting or repelling agents to/from each other. For example, golden-crowned sparrows (*Zonotrichia atricapilla*) establish dominance via aggressive contests in their winter ranges. Although more dominant sparrows often exclude subordinates from food sources, familiar pairs that have an established dominance relationship have fewer aggressive interactions than two unfamiliar sparrows [33]. Aggression, tolerance and familiarity interact with exogenous factors such that individuals within populations become differentiated. Among Asian elephants (*Elephas maximus*), in which the FF process itself undermines the formation of dominance hierarchies, conflicts can be avoided through spatial distancing [67].

Heterogeneity in the environment gives structure to potential dispersal paths and thus influences larger scales of the association at the population or even meta-population level. At higher spatial scales, environmental features such as mountains, rivers, water currents, anthropogenic activities and other physical barriers can cause populations to become separated [68]. Barriers reinforce social connectivity within populations and *discontinuity* among populations. For example, landscape-level barriers to gene flow can lead to increasing relatedness between individuals within a population, which then strengthens links between genes and social associations, reinforcing social structure at multiple levels of organization [32,69]. Thus, kinship patterns at these large spatial scales are likely to be driven much more by exogenous features of the environment than endogenous factors, which can again feed back to the smaller spatial scales.

## 4. Social scale

Dyadic structures (i.e. presence, absence and strength of connections) represent pairwise interactions between agents. Processes at this organizational scale include the development of relationships, maintenance of existing relationships and dissolution of relationships. Agents may consistently and preferentially associate with each other owing to endogenous benefits, such as fewer aggressive encounters, reduced stress or pair bonds (e.g. [33]). Other endogenous factors like demography influence patterns of connections when individuals show assortment based on kinship, sex or social phenotypes [70,71], or when nodes are added or removed via demographic turnover [72]. Exogenous factors such as resource distribution and availability indirectly influence pairwise interactions when pairs consistently visit the same areas (increased strength) or when there are pairwise mismatches in motivation or ability (decreased strength) [73].

**Figure 3.**
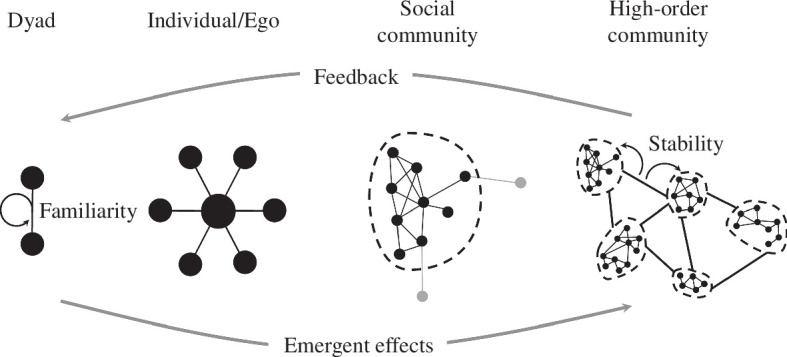
Nested social scales. Social organization emerges from interactions between agents at lower scales. Communities (dashed lines) may vary in permeability. Feedbacks and interactions within and between social scales can reinforce or perturb social structure and organization.

Community-scale structure and organization emerge from dyadic interactions (figure 3). Here, we emphasize a distinction that has seldom been remarked upon, which is that social communities can either be clearly bounded or not, and may be so at different scales [74,75]. This distinguishes, for instance, social systems such as that of chimpanzees from that of all elephants, and that of African savannah elephants from that of Asian elephants. Chimpanzees can be considered high-FF relative to African savannah elephants in that foraging parties are much more fluid than family groups of savannah elephants; FF occurs at the level of individuals in chimpanzees and at the level of families in savannah elephants. However, chimpanzee communities have clear territorial boundaries, which when crossed can elicit fatal attacks from neighbouring communities [76]. This is not so with savannah elephants, where family groups easily co-mingle with one another even if more dominant families can enjoy priority of access to key resources [77]. Therefore, at the larger social scale of multiple communities, savannah elephants exhibit greater FF than chimpanzees. African savannah elephant families, however, are cohesive enough that they are easily identifiable as both social and spatial units, even if not territorial. Not so with Asian elephants, where spatiotemporal associations change not only on day-to-day timescales but also over longer seasonal and annual timescales [78,79]. FF again occurs at the level of individuals and is higher, with social units even less clearly bounded in Asian elephants than in savannah elephants. This should not, however, obscure the observation that individuals do selectively affiliate with other individuals with whom they develop a history over their lifetimes, rather than randomly mixing with the entire population, and that there can be variation even among populations of the same species [40,79].

Larger social scales, which may correspond to longer temporal scales, larger spatial scales or both, encompass population and even meta-population FF dynamics. The agent need not be aware of these global structures; however, in times of disturbance, their memories of individual interactions can help to maintain and reconstitute the larger networks [7,80]. This type of emergent pattern can be termed *resilience*, reflecting a system’s ability to maintain (or regain) core functions and structures despite perturbations. For example, animal social systems may have high resilience to changes in lower-level structural or organizational components, such as demographic turnover events, but only up to a critical *threshold* and not to changes to key components, such as agents that act as ‘hubs’ that connect across the entire network or agents that play key social roles [81,82].

Multi-scalar social structure has been well-studied in mammalian and avian systems that show discrete levels of organization [7,50,83,84], leading to many insights about how hierarchical structures emerge from lower-level interactions. Nested networks—a type of multi-layer network sometimes also referred to as ‘networks of networks’ [85]—are a useful way of differentiating among scales [78,86,87]. Nested networks can incorporate interdependent processes that occur across organizational scales, while also describing dynamics within scales [88]. Some nested networks may be grouped into very clear, hierarchical and discontinuous scales of organization, but not all. It has been proposed that the term ‘multi-level’ should be limited to societies that have clearly defined and stable core units as well as discontinuities among levels [16]. This restricts it to a relatively small subset of social species. However, social networks may not always exhibit discrete levels of organization, although individuals can and do differentially associate with one another to varying degrees. As recognized early on by Caceres & Berger-Wolf [44], the special challenge of dynamic networks is that phenomena that we presume to label as biologically meaningful, including social units themselves, may be moving targets. Group size and composition, modularity and scales of *social organization* are dynamic properties in social networks, therefore ‘multi-level societies’ are better thought of as a very narrow subset of multi-scalar social systems.

This distinction is especially prominent if we consider clades that are greatly neglected in FF literature because they may be cryptic in behaviour and/or do not easily conform to mammalian, avian or arthropod-based notions of ‘social’ groups, such as reptiles [89]. North American pit vipers (*Crotalus atrox*), for instance, can live up to 60 years and hunt solitarily but vary their associations during communal winter denning, mating and offspring production [90]. Individuals show extremely high fidelity to denning sites over multiple years but denning networks show low genetic relatedness and females are less likely to den communally than males. Individuals are not limited to one set of associates but make state-dependent switches among different subsets of the population to form modular, but not nested, networks. CAS accommodate such dynamics and encourage us to seek more examples of cases that do not fit classical social models, which may be more widespread than we currently recognize.

## 5. Conclusions

As biologists, we have a fondness for classifying things, be they cellular components, organisms, societies or ecosystems. This has served us well in general but causes discomfort with parts of the natural world that defy rigidity, such as species hybridization zones or disturbance regimes that give rise to multiple alternative ecological states. CAS encourage us to embrace the messiness. Aureli *et al.* [2] took early steps in this direction by pointing out that there is no such thing as a ‘fission–fusion social system’, and instead that these processes can be exhibited to varying degrees by many different types of systems. Sueur *et al.* [3] proposed these dynamics to be modelled on the basis of individual decisions. The CAS approach is a logical extension of this way of thinking and is more than just a different way of describing the same phenomena. It pushes us to give primacy to the dynamics of the system and treat social groupings of various kinds as emergent phenomena, even if they are transient.

This does not mean that we dispense with socioecological theories or models, only that we explicitly acknowledge their limits at various scales and differentiate exogenous from endogenous sources of change without disproportionately emphasizing one or the other. It also encourages us to frame different questions, such as: at what scales and under what conditions does social stability occur? How do switch points help us identify changes in stability, resilience or state space of FF dynamics? When do FF dynamics result in social groupings that adhere to traditional socioecological models and when do they diverge owing to self-organization and updation at lower levels? Do responses to simulated disturbances match reality, and if not, why? Understanding the potential for changes that are inherent in animal social systems is important for establishing a baseline for the social dynamics we expect under a given set of conditions, and how they may react to stresses. With established baselines, we may identify variables that act as an early warnings of decreasing resilience or the start of a state shift [21]. For example, the loss of community structure may signal a breakdown in dyadic connections from widespread demographic change (endogenous perturbation) or a sudden loss in landscape connectivity (exogenous perturbation). This has important implications for the study of the evolution of social systems as well as the conservation and management of social species. Future research can expand this framework even further to incorporate additional components or redefine the boundaries of the system, for example, to include cases of interspecific sociality and ecosystem feedback.

## Data Availability

This article has no additional data.
